# Isolated Spin-7/2 Species of Gadolinium (III) Chelate Complexes on the Surface of 5-nm Diamond Particles

**DOI:** 10.3390/nano13131995

**Published:** 2023-07-01

**Authors:** Vladimir Yu. Osipov, Danil W. Boukhvalov, Kazuyuki Takai

**Affiliations:** 1 Ioffe Institute, Polytechnicheskaya 26, St.-Petersburg 194021, Russia; 2 Institute of Materials Physics and Chemistry, College of Science, Nanjing Forestry University, Nanjing 210037, China; danil@njfu.edu.cn; 3 Institute of Physics and Technology, Ural Federal University, Mira 19 Str., Yekaterinburg 620002, Russia; 4 Department of Chemical Science and Technology, Hosei University, Tokyo 184-8584, Japan; takai@hosei.ac.jp

**Keywords:** detonation nanodiamonds, gadolinium, surface functionalization, paramagnetism, magnetization, electron paramagnetic resonance

## Abstract

The magnetic characteristics of a system of triply charged gadolinium ions Gd^3+^ chelated with carboxyls on the surface of detonation nanodiamond (DND) particles have been studied. Gd^3+^ ions demonstrate almost perfect spin (S = 7/2) paramagnetism with negligible antiferromagnetic interaction between spins (Weiss temperature about −0.35 K) for a wide range of concentrations up to ~18 ions per 5 nm particle. The study of the concentration dependence of the electron paramagnetic resonance signal for DND intrinsic defects with spin ½ (*g* = 2.0027) shows that Gd^3+^ ions are located on average at a distance of no more than 1.4 nm from shallow subsurface defects with spin ^1^/_2_. At the same time, they are located (according to density functional theory calculations) at a distance of about or at least 0.28 nm from the particle surface. Magnetic studies also confirm the isolated nature of the gadolinium chelate complexes on the surface of DND particles. DND particles turn out to be an optimal carrier for high-spin *4f-* ions (gadolinium) in a highly concentrated isolated state. This property makes DND-Gd particles a candidate for the role of a contrast agent for magnetic resonance imaging.

## 1. Introduction

Detonation nanodiamonds (DND) have been known to the scientific community since the late 1980s [1]; however, they became a genuinely new carbon material only at the end of the 2000s [2], when ample opportunities opened up for the preparation of monodisperse aqueous DND suspensions with a particle size of about 5 nm [3] and their functionalization with various atomic groups and complexes [4]. The methods developed then made it possible, among other things, to obtain DND particles with a predominantly carboxylated surface.

The possibility of functionalizing the surface of 5-nm particles of carboxylated detonation diamond with *3d*- and *4f*- transition metal ions forming chelate complexes has been known in principle for a long time [5]. This possibility is realized due to the ion exchange of metal cations with protons of carboxyl groups on the surface of particles in an aqueous medium, due to which doubly charged cations are captured by pairs of carboxy groups, and triply charged ones, in turn, by pairs or triplets of carboxy groups. Among *3d*- metals, the best agent that clings to the surface of DND particles is bivalent copper, where the source of such copper is copper (II) nitrate [6]. Careful magnetic studies performed in 2010–2022 showed that doubly charged copper is fixed on the surface of 5-nm particles in an amount of up to 18–20 ions per DND particle [6]. This value indirectly indicates the presence of at least ~40 COO^−^ groups located on the surface, actively participating in the binding of copper cations. Such sites are only 3–3.5% of the total number of carbon atoms on the surface with outward-facing σ-bonds. Previous estimates show that a partially carboxylated (by 30–35%) DND surface has, in principle, a large sorption capacity and can accommodate up to ~200 doubly charged metal ions [7,8]. Among the *4f*-metal cations that successfully attach to the surface of carboxylated DND, there is also the gadolinium ion Gd^3+^. The first studies on the fixation of Gd^3+^ on the DND surface with the formation of the corresponding chelate complex were published by us in 2015 [9]. The Gd^3+^ ion has a large magnetic moment with spin *S* = 7/2 and zero orbital quantum number *L* = 0 and, therefore, can be easily revealed by classical magnetic methods by measuring the temperature dependence of the magnetic susceptibility of the hybrid material or by measuring magnetization curves with saturation at low temperature (usually helium). Interest in DND particles functionalized with gadolinium ions (via molecular linkers of various lengths, including hyperbranched ones) is due to the fact that such hybrid nanoparticles can be used to enhance the image contrast in magnetic resonance imaging (MRI) [10,11,12]. The cause of this enhancement is the following: atoms with large magnetic moments significantly reduce the time of magnetic relaxation of protons of water molecules, as a result of which the morphological features of tissues in places where there is an increased accumulation of particles with ferromagnetic atoms due to sorption are easily visualized. The higher the local concentration of ferromagnetic atoms on such particles, the stronger the contrast in MRI images can, in principle, be achieved. Indeed, the use of DND-Gd^3+^ particles as a contrast agent for MRI imaging has recently been reported in [13].

The behavior of Gd^3+^ ions on the surface of DND particles has not yet been sufficiently studied regarding the presence (or absence) of weak ferromagnetic or weak antiferromagnetic interaction between ions on the surface of DND particles [9]. In the case of isolated Gd^3+^ ions on the surface of DND particles fixed through carboxyl groups (the absence of gadolinium atoms in the metal cluster form is implied), and in the absence of *sp*^2^-conducting fragments on the surface, ferromagnetic interaction between the ions should theoretically be absent. The material should demonstrate paramagnetic properties for the for the 7/2 spin ensemble. The purpose of this work is to test the magnetic status of an ensemble of Gd^3+^ ions on the surface of DND particles in the case of an extremely high concentration of gadolinium in the material (up to 2 wt %) and to elucidate the question of the possible superparamagnetism of such hybrid particles. In this case, the magnetic status of the ions can be checked by analyzing experimental data of various types: obtained from the magnetization curves at the minimum achievable temperature in the experiment, and from the analysis of the temperature dependence of the magnetic susceptibility of the material measured at a fixed value of the magnetic field in the linear section of the dependence of the magnetization on the magnetic fields.

The main difficulty in assessing the magnetic properties of Gd^3+^ ions on the surface of DND particles is the need for a methodologically correct exclusion from consideration of the inherent paramagnetism of DND associated with paramagnetic defects [14] of the diamond matrix and the weak diamagnetism of the diamond matrix [15]. Another parallel goal of this work is the use of gadolinium spins as paramagnetic probes with a large magnetic moment to determine the depth of shallow paramagnetic centers in the DND crystal lattice.

## 2. Samples and Research Methods

Powder samples of DND particles with a surface functionalized with Gd^3+^ ions were prepared by mixing an aqueous suspension of DND with a negative zeta potential with an aqueous solution of gadolinium nitrate and subsequent isolation of the solid product according to the procedure described in [9]. Gadolinium nitrate hexahydrate Gd(NO_3_)_3_·6H_2_O and a DND suspension diluted to 0.25 wt % with pH = 7.3 were chosen as precursors. The synthesized samples with different nominal contents of gadolinium (within almost two orders of magnitude) are further designated as DG1, DG2, DG3, and DG4. The original DND powder unmodified by metals (zero Gd^3+^ concentration) is designated as DG0. A series of samples with different concentrations of gadolinium was studied by SQUID magnetometry and electron paramagnetic resonance. In the first case, powders weighing up to 75–77 mg were packed into aluminum foil capsules and placed in the middle part of Pyrex tubes (inner diameter 5 mm, length 200–220 mm). The weight of an aluminum capsule made from thin foil (cut from one part of the roll for all samples) averaged 51–58 mg. 

The tubes were evacuated to a high vacuum (2 × 10^−6^ Torr) at room temperature and sealed on both sides using a gas burner. To eliminate the influence of temperature effects on the arrangement of functional groups on the surface of the samples, the temperature of sample pretreatment in a vacuum did not exceed room temperature. The sample tube prepared in this way was placed in the measuring chamber of an MPMS-7 SQUID magnetometer (Quantum Design, San Diego, CA, USA). The temperature dependence of the *dc* magnetization of the sample in the capsule was measured in a field of 10 kOe in the temperature range of 2–300 K, as well as the magnetization curve of the sample at a temperature T = 2 K in a field ranging from zero to 70 kOe. The temperature in the measuring chamber was maintained with an accuracy of ±0.02 K, while the temperature sensor was located near the chamber wall. Before each measurement, carried out with a change in temperature, the sample was kept in the chamber from 120 to 180 s to stabilize and equalize the temperature throughout the entire volume of the sample and the tube holding it. The time interval between the fixation of the magnetic field and the time of the beginning of the magnetization measurement was 60 s. The magnetization was measured by vertical scanning of the sample in the tube relative to measuring superconducting coils (turns), which are the antenna of the SQUID sensor. The number of scans was chosen to be 3. To assess the magnetic characteristics of the DND-Gd^3+^ powder itself, the value of the magnetization corresponding to the weight of the aluminum foil capsule was subtracted from the total magnetization measured for each value of the magnetic field and temperature. The magnetic characteristics of the used aluminum foil were recorded in advance and recalculated per unit weight for the convenience of subsequent subtraction of the contribution from the capsule. In practice, the temperature course of the susceptibility of aluminum foil was recorded once for a sample weighing 234 mg, cut from the middle of the roll, and then each time recalculated for the weights of individual Al containers cut from the same place in the roll and containing powder samples DG1–DG4. 

After subtracting the contribution from the magnetization of the foil, the found magnetization of the powder was assigned to the unit weight of the synthesized product. The magnetic susceptibility *χ*, expressed in units of *emu g*^−1^ (in the CGS system), was determined by the formula *M*/*H*, where *M* is the specific magnetization of the powder under study, and *H* is the magnetic field strength in Oe. For example, for the specific magnetization of a sample of 1 × 10^−2^ emu g^−1^ measured in the field *H* = 10,000 Oe, the magnetic susceptibility is 1 × 10^−6^ emu g^−1^ (oersteds are often not specified in units of magnetic susceptibility, written in the CGS system). Using oersteds as the units of the magnetic field, the magnetic susceptibility can also be written in the format 1 × 10^−6^ emu g^−1^ Oe^−1^. The EPR spectra of the samples were recorded using a JEOL JES-FA 300 X-band EPR spectrometer (JEOL Ltd., Tokyo, Japan) at room temperature. The recorded main EPR signal was decomposed into two Lorentzian signals (narrow and broad) with close g-factors, for each of which its width *ΔH_pp_* was found. For the narrow and broad components of the EPR signals, the dependence of the linewidth on the concentration of Gd^3+^ ions was analyzed.

The structure of gadolinium chelate complexes in the “dry” state (without additional water molecules coordinating metal atoms) was calculated by the electron density functional theory (DFT) using a pseudopotential program code SIESTA [16]. The calculations were performed in the generalized gradient approximation (GGA-PBE) with spin polarization [17] using corrections for van der Waals forces [18]. To model a fragment of a diamond particle with a (111) surface, we used a piece of a crystal lattice of four carbon layers, passivated on one side by three closely spaced carboxy groups and hydrogen atoms. The calculated structures were further visualized in the Mercury 2.3 program (Cambridge Crystallographic Data Centre, Cambridge, UK) to determine characteristic distances and provide visualization of the most informative views. 

## 3. Experimental Results

### 3.1. Magnetization Curves and Magnetic Susceptibility Data 

**Figure 1:**
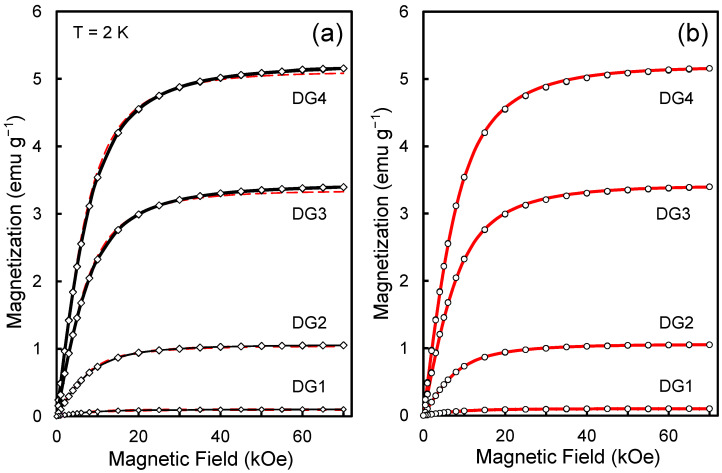
M vs. H plots for a series of Gd-grafted DND samples at 2 K with two series of fitting curves described by conventional Brillouin functions (**a**) and temperature-corrected Brillouin functions in argument (**b**). The second set of Brillouin curves shown in panel (**b**) considers the Weiss temperature correction in the framework of the Weiss molecular field theory. Fitting Brillouin curves: dashed red lines (**a**) for the standard *B*- function and continuous red lines (**b**) for the function with corrected argument. The continuous black lines from panel (**a**) simply connect the experimental points.

The dependences of the magnetization of ensembles of Gd^3+^ ions on the applied magnetic field at T = 2 K are shown in Figure 1a for each of the samples of the DG1-DG4 series. They were obtained by subtracting the magnetization of the initial unmodified DND powder (DG0) from the overall magnetization of the gadolinium-modified DND powder (DG1…DG4). Note: here and below, we mean the specific magnetization, i.e., the magnetization of the sample (modified or unmodified) divided by the weight of the powder (or carbon matrix). The magnetization curves (*M*-*H*) recorded for the forward and reverse sweep of the magnetic field according to the 0→ +70 kOe →0 scheme did not reveal significant hysteresis and noticeable residual magnetization of the sample in zero field.

Fitting these curves with the help of functions described by the Brillouin formula for spins S = 7/2 and T = 2 K made it possible to determine the concentration of Gd^3+^ ions for each of the samples in the series. Here, the concentration of gadolinium ions *N*_Gd_ acts as the main fitting parameter. Recall that the magnetization of an ensemble of localized spins at low temperatures, determined by the average projection of the magnetic moments of the spins on the direction of the magnetic field **H**, is described by the Curie-Brillouin law [19,20,21]:

(1) $$M=N_{Gd}gS\mu_{B}\cdot \left[\frac{2S+1}{2S}\coth\frac{(2S+1)x}{2S}-\frac{1}{2S}\coth\frac{x}{2S}\right]$$

where $x\equiv gS\mu_{B}H/k_{B}T$.

Here *T* is the temperature, *k_B_* is the Boltzmann constant, *μ_B_* is the Bohr magneton, *S* is the spin quantum number of the paramagnetic center (or *4f*- ion) prevailing in the system and responsible for paramagnetism in it (note: for the gadolinium ion Gd^3+^, Formula (1) contains not the total angular momentum quantum number *J*, as for most transition *4f*- ions, but the spin *S*, since for this ion the orbital angular momentum quantum number is zero (*L* = 0) and the quantum number *J*, determined by the sum *L* + *S* is equal to *J* = *S*), *g* is the Lande factor or gyromagnetic multiplier equal to 2 in our case, *N*_Gd_ is the concentration of isolated paramagnetic centers (in our case, Gd^3+^ ions) with spin *S*, and the factor in square brackets is the Brillouin function describing the trend with saturation with increasing dimensionless parameter *x*. Note that Formula (1) is written in the Gaussian system of units (CGS) when the relationship between the magnetic field *H*, magnetic flux density *B* and magnetization *M* of the material is written as B=H+4πM [20]. In this notation, unlike that in the SI system of units, the magnetic constant $\mu_{0}$ is not used (in fact, it is 1), and the dimension of the unit for the magnetic field strength *H* (Oe) is same as that for magnetic flux density *B* (G). Recall that the derivation of this formula assumes Boltzmann statistics for an ensemble of magnetic moments of spins, and the ensemble itself consists of noninteracting identical magnetic moments. In weak fields or at high temperatures, when x<<1, the formula is simplified and we have $M=\left(N_{Gd}g^{2}\mu_{B}^{2}S(S+1)/3k_{B}T\right)\times H$, i.e., the linear dependence of *M* on *H*. The linear dependence of *M* on *H* is well observed in fields up to 4.5 kOe in the entire temperature range, including the low-temperature region (*T* = 2–5 K). 

The values of $N_{Gd}$ obtained from the experimental data are given in Table 1 (second column). The high degree of agreement between the experimental and theoretical curves shown in Figure 1a indicates that the system does indeed contain magnetic agents with spin *S* = 7/2 in concentrations from 1.53 × 10^18^ g^−1^ to 7.85 × 10^19^ g^−1^, and the contributions from magnetic units with other spins are practically absent. Better fitting of experimental curves using Brillouin functions can be achieved by varying the actual temperature *T* of the sample in the chamber in a small range (from 2 to 2.25 K). The best fittings are achieved at *T*_eff_ = 2.10–2.19 K (Figure 1b), which is reasonable, taking into account the possible difference in temperature on the wall of the measuring chamber at the place where the *T*-sensor is located from the actual temperature of the sample in a massive Pyrex tube, reciprocating within 60 mm inside the chamber. This, however, has almost no effect on the corrected estimates of the *N*_Gd_ values for all the samples under study. The concentrations of isolated Gd^3+^ ions, obtained considering the corrected temperature *T*_eff_ = *T*_sensor_ + Δ*T* (in the range of 2–2.25 K), corresponding to the “warmer” sample, are presented in the third column of Table 1. The fourth column of Table 1, in turn, shows the values of Δ*T.*

**Figure 2:**
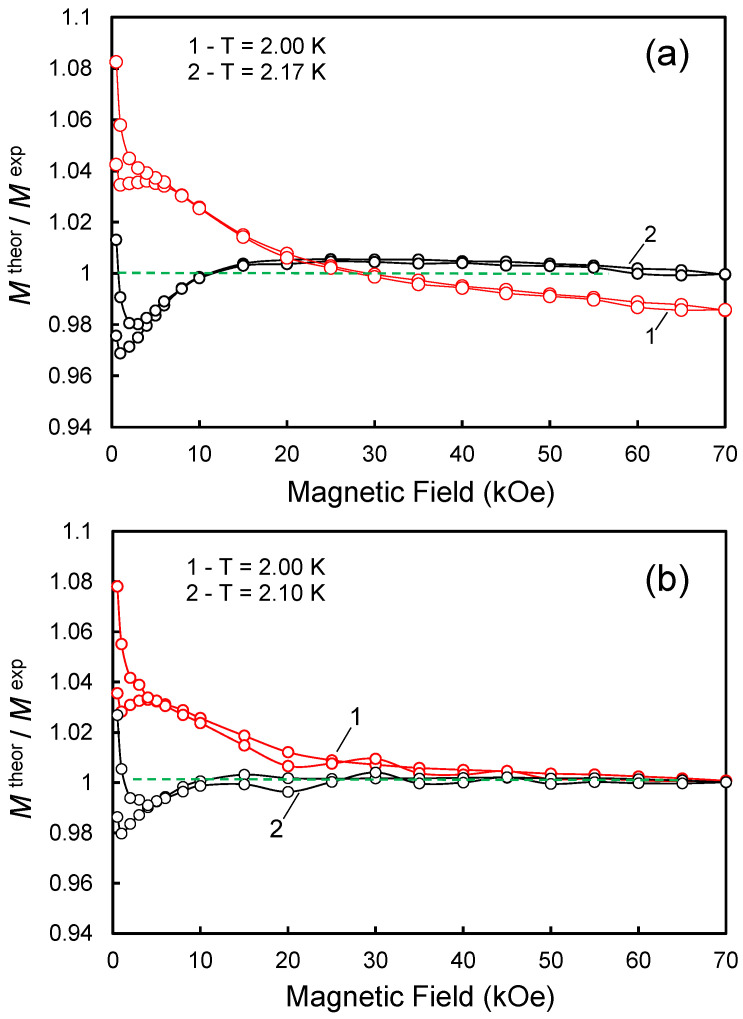
Dependence of the ratio *M*^theor^/*M*^exp^ on *H* at *T* = 2 K for two methods of approximation of the magnetization curve by Brillouin functions, designed both without (curve 1) and taking into account (curve 2) the addition of Δ*T* to the nominal temperature *T* in the function argument *x*. Panel (**a**): sample DG4, 1 − Δ*T* = 0*;* 2 − Δ*T* = 0.17 K. Panel (**b**): sample DG2, 1 − Δ*T* = 0*;* 2 − Δ*T* = 0.10 K.

In summary, the fitted magnetic curves obtained with corrected temperature *T*_sensor_ + Δ*T*, where Δ*T* < 0.25 K, pass through the experimental points better. As a criterion for the perfection of such fitted curves, one can choose a measure of the deviation of the ratio of the theoretical value of magnetization (*M*^theor^) to the experimental value of magnetization (*M*^exp^) from 1 in the entire range of applied magnetic fields from 0 to 70 kOe. Thus, Figure 2a shows the magnetic field dependence of the ratio (*M*^theor^/*M*^exp^) for two fittings made with temperatures T = 2 K and T = 2.17 K for the DG-4 sample with the maximum content of Gd^3+^ ions. Each of the presented dependences *1*, *2* is plotted for two directions of a slow magnetic field sweep (in the range from zero to the maximum field of 70 kOe and from the maximum field to zero). It can be seen from these dependences that the approximation by the Brillouin function at an effective temperature of 2.17 K gives better results in a wide range of magnetic fields with an accuracy of ±3%, and the largest relative deviations from the theoretical dependence occur in the field range up to 8 kOe, where weak hysteresis effects are manifested. Figure 2b, in turn, shows the dependence (*M*^theor^/*M*^exp^) vs. *H* for the DG2 sample with a relatively low content of Gd^3+^. In this case, the effective temperature, which gives the best agreement between the theoretical and experimental data, is 2.10 K. It can be seen that in the case of the low-concentration Gd^3+^ system of sample DG2, the ratio (*M*^theor^/*M*^exp^) is close to 1 within 8–70 kOe with an accuracy of ±0.5%, and the magnetization curve is approximated better by the Brillouin function in the entire range of magnetic fields than in the case of a highly concentrated system DG4. However, in fields *H* < 6 kOe, small (up to 2%) deviations from the experimental magnetization *M*^exp^ are still present. This, however, is not essential for further analysis.

It is possible to explain the appearing Δ*T* as an addition to the nominal temperature of the sensor, taking into account the weak interaction between the spins. In this case, the Brillouin formula has a modified form and is written with the argument $x\equiv gS\mu_{B}H/k_{B}(T+∆T)$, where Δ*T* is already a term in the denominator that considers the exchange interaction [22]. More precisely Δ*T* = − *Θ*, where *Θ* is the Weiss temperature. The occurrence of the term T−Θ in the argument *x* is associated, as is known from the Weiss theory, with the addition of the molecular field $H_{m}$ to the magnetic field *H* in Formula (1), where $H_{m}$ is proportional to the magnetization of the sample *M*: $H+H_{m}=H+\lambda M=H\frac{T}{T-\theta }$. Here λ is the Weiss theory’s molecular field constant proportional to Θ. In this case, for all DG1-DG4 samples under study, we have a weak antiferromagnetic interaction between the Gd^3+^ spins, the value of which does not exceed 0.25 K.

**Figure 3:**
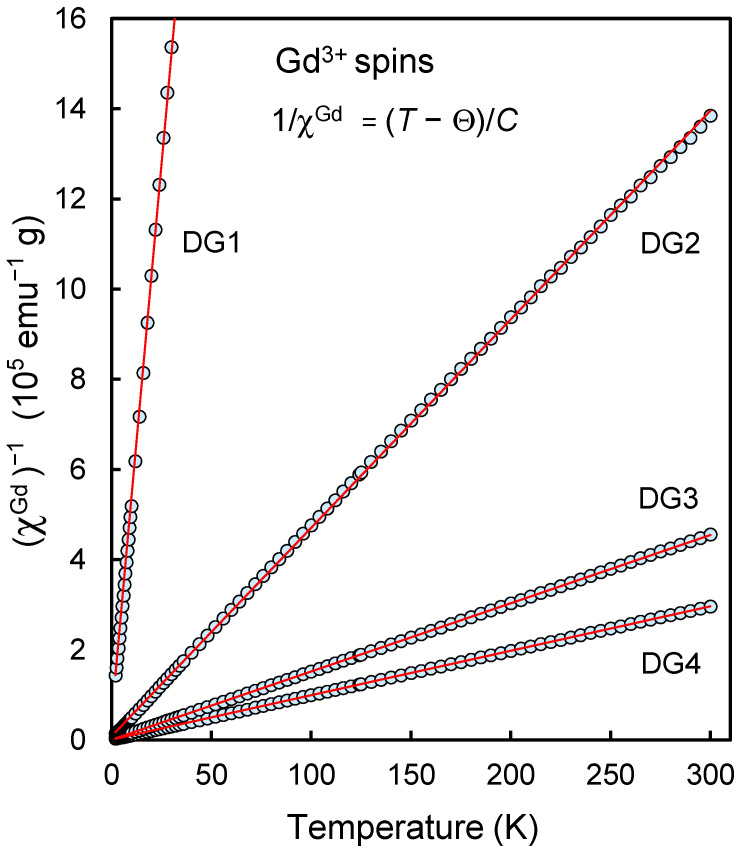
Temperature dependence of the inverse magnetic susceptibility of the Gd^3+^ ion system on the DND surface for the series of DND-Gd samples under study with different Gd^3+^ concentrations. The measurements were performed in a field of 10 kOe when the sample was heated from *T* = 2 K to room temperature. The contribution to the magnetic susceptibility from the paramagnetism and diamagnetism of the carbon subsystem was correctly subtracted at the stage of data processing.

Figure 3 shows the temperature dependencies of the magnetic susceptibility of the ensemble of Gd^3+^ ions of samples DG1-DG4 plotted in coordinates (*χ*^Gd^)^−1^ versus *T*. To obtain such dependences, from each experimental dependence *χ*(*T*) obtained for each of the samples DG1…DG4, we subtracted $\chi^{DND}(T)$, the corresponding temperature dependence of the susceptibility for the initial gadolinium-free sample DG0. It is assumed that the contributions to the total magnetization from the components associated with the ensemble of Gd^3+^ ions on the surface of DND particles and the ensemble of spins with S = ½ inside the diamond lattice are additive. That is, the fixation of Gd^3+^ ions on the surface of DND particles does not lead to recharging and a change in the magnetic status of spins with S = ½ of the intrinsic defects of the diamond lattice, which determine the intrinsic paramagnetism of DND particles. It should also be emphasized that the dependence of the magnetic susceptibility $\chi^{DND}(T)$ on temperature for the initial DND sample, in addition to the temperature-dependent positive paramagnetic component $\chi_{CW}$, associated with spins ½ inside the diamond matrix and obeying the Curie-Weiss law, also contains a temperature-independent the negative component $\chi_{0}$ associated with the intrinsic diamagnetism of the covalent *sp^3^*- coordinated diamond lattice. Both of these components are essential when analyzing the magnetic characteristics of DNDs with a low gadolinium content, for example, less than one or two Gd^3+^ ions per 5-nm DND particle. 

Therefore, the correct subtraction of the intrinsic magnetic susceptibility of a nanodiamond from the susceptibility of a modified DND is an essential procedure for determining the magnetic susceptibility of an ensemble of Gd^3+^ ions. For the magnetic susceptibility of an ensemble of Gd^3+^ ions, we have the following formula:

(2) $$\chi^{Gd}=\chi^{exp}-\chi_{CW}^{DND}\left(T\right)-\chi_{0}^{DND}$$

here, the first term $\chi^{exp}$ is the experimentally measured magnetic susceptibility of the hybrid nanomaterial, and the second and third terms are, respectively, the contributions to the initial DND susceptibility from the Curie paramagnetism of the intrinsic spins S = ½ of diamond nanoparticles and the diamagnetism of the diamond lattice. In our case, for DND, the value of $\chi_{0}^{DND}$ is approximately –(0.36 ± 0.03) × 10^−6^ emu g^−1^. The values of $\chi_{0}^{DND}$ determined experimentally for each of the samples of the series are given in the rightmost column of Table 1. It should be noted here that the value of $\chi^{Gd}$ is not strictly the specific magnetic susceptibility of gadolinium atoms, in which the magnetization of gadolinium atoms is related to the unit of magnetic field and unit weight of gadolinium atoms, but the value referred for ease of analysis to the unit weight of carbon atoms of the matrix. The temperature dependences $\chi^{Gd}(T)$ thus obtained were further analyzed as inverse functions of temperature.

**Figure 4:**
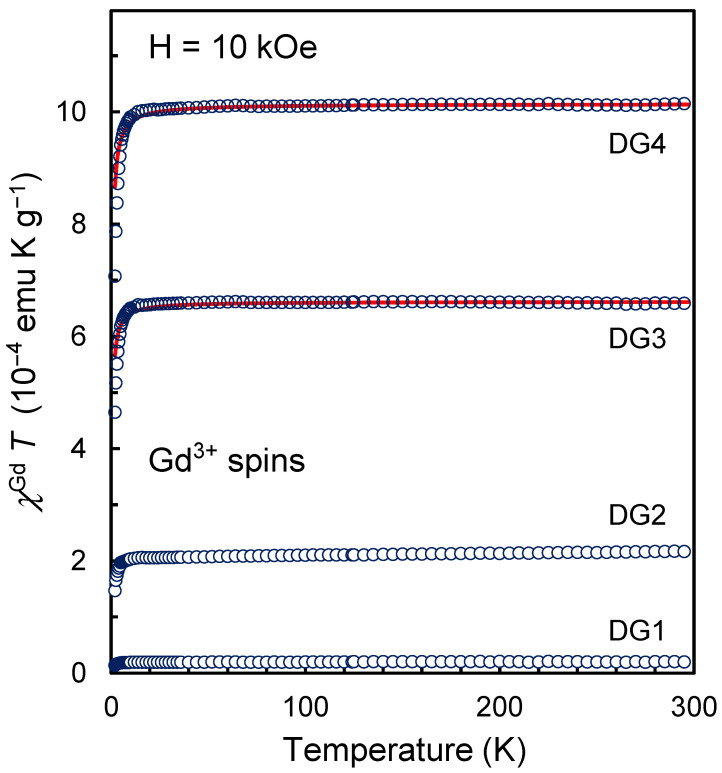
Temperature dependence of the product $\chi^{Gd}$*T* for a system of gadolinium ions Gd^3+^ on the DND surface for DND-Gd samples with different concentrations of Gd^3+^. The contribution to the magnetic susceptibility from the carbon subsystem was subtracted at the data processing stage. The red lines here show the theoretical approximations by the CT/(T−θ) functions, where *Θ* < 0 is an adjustable parameter describing the low-temperature downward bending of the functions. The fitting parameters *Θ* for both red curves are −0.34 K.

The curves presented in Figure 3 are linear in the ${1/\chi }^{Gd}$ vs. *T* coordinates in the range of 2–300 K and reflect the fact that the ensemble of Gd^3+^ ions for all the studied samples demonstrates pure paramagnetism and with high accuracy (especially at *T* > 5 K) follows the Curie law: $\chi^{Gd}=\frac{C}{T-\Theta }$, here *C* is the Curie constant associated with the concentration of spins S = 7/2 of gadolinium ions in the ensemble, and *Θ* is the Weiss temperature characterizing the magnetic interaction between the spins of the complexes, are parameters determined from the slope of the curve and the cutoff along the temperature axis. In other words, Weiss’s mean-field parameter λ introduced above is an inter-complex interaction factor *T*/(*T* − *Θ*). Table 1 (sixth column) shows the values of parameters *C* obtained from the slopes of the corresponding dependences ${1/\chi }^{Gd}$ vs. T for the series of samples under study. The *C* value is related to the spin concentration $N_{Gd}^{T}$ as follows: $C=\frac{\mu_{B}^{2}}{3k_{B}}N_{Gd}^{T}g^{2}S(S+1)$, here almost all constants and variables ($\mu_{B}$, *g*, *S*, $k_{B}$) from the formula have already been defined above. Table 1 also shows the values of the parameters $N_{Gd}^{T}$ and *Θ* for each studied sample. Small negative values of *Θ* (from −0.69 to −0.33 K), obtained by extrapolating the fitting straight line passing through the experimental points to the value of ${1/\chi }^{Gd}$ =0, indicate that the magnetic interaction between spins *S* = 7/2 of Gd^3+^ ions or between surface spins 7/2 and underlying spins ½ of the carbon matrix is antiferromagnetic and very weak even for the highest realizable concentration of gadolinium ions in the system. As can be seen from Table 1, the values of $N_{Gd}^{T}$ and $N_{Gd}^{M-H}$ coincide with each other with reasonable accuracy. Additionally, Figure 4 shows the dependences $\chi^{Gd}T$ vs. *T*. For all four studied samples, the value of $\chi^{Gd}T$ is decreased by ~30% (from the corresponding value at 100 K) when the temperature is lowered to the minimum achievable. This decrease is realized in the temperature range below 10 K. The formal reason for this is related to the presence of the value *Θ* in the formula for the temperature dependence of $\chi^{Gd}$ and the corresponding features of the function CT/(T−θ) when approaching the minimum achievable temperature of about 1.98–2.02 K. Analysis of the data from Figure 4 gives a range of possible values of *Θ* from −0.35 to −0.34 K for all samples. This confirms the above statement about a very weak antiferromagnetic coupling in the system of gadolinium spins 7/2. Thus, lowering the temperature to 1.98–2.02 K does not lead to the formation of antiferromagnetically coupled Gd…Gd or Gd…R* pairs in a noticeable amount, as, for example, happens for binuclear metal complexes and Gd…R* complexes based on gadolinium and radicals of different nature [23], and a decrease in the effective concentration of spins 7/2 on the surface of DND particles. This at least means that the Gd^3+^ ions recorded by magnetic methods are located on the surface of the DND particle in isolation and as far as possible from each other. Consequently, the system lacks both antiferromagnetism and superparamagnetism from *4f-* elements on the surface of nanoparticles. The ensemble of Gd^3+^ ions acts as a set of almost magnetically noninteracting spins S = 7/2. The concentration of Gd^3+^ ions found by two methods in the DG4 sample is on average 7.86 ^(±0^.^11)^ × 10^19^ g^−1^ which corresponds to about 18 Gd^3+^ ions on the surface of a diamond particle with a diameter of 5 nm and a weight of ~2.3 × 10^−19^ g. In other units more familiar to materials scientists, this concentration of Gd^3+^ ions for sample DG4 is ~1570 ppm. It is assumed here that the weight of the molecular shell ~0.23 nm thick is included in the particle’s mass. The ratio [Gd]/([C] + [O] + [H]) is 1.56 × 10^−3^ in this case. The concentration of gadolinium ions of the order of ~20 pieces on the surface of a sphere 5 nm in diameter makes such an object potentially interesting for obtaining hybrid particles that accelerate the relaxation of the magnetic moments of water protons and improve the contrast in magnetic resonance imaging.

### 3.2. DFT Modeling and Layout Topology of Gd^3+^ Ions

**Figure 5:**
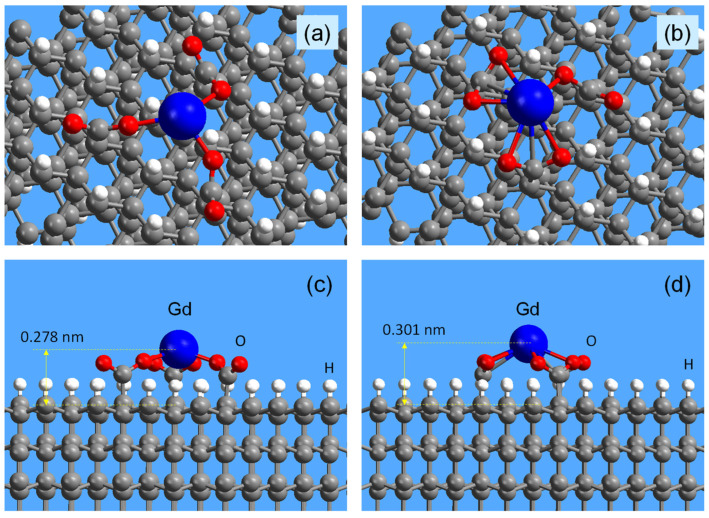
Configurations 1 and 2 of Gd^3+^ chelate complexes on the (111) surface of diamond, calculated by the DFT method: (**a**,**c**) configuration 1; (**b**,**d**) configuration 2; (**a**,**b**) top view slightly at an angle to the (111) plane of the diamond; (**c**,**d**) side view. Atoms are shown in the following colors: gadolinium—blue, oxygen—red, carbon—brilliant gray, and hydrogen—white.

Chelate complexes of gadolinium can exist on the diamond surface in different configurations. However, they are all basically the same if they are bound to the surface by two or three carboxy groups. The unsaturated coordinations of the gadolinium ion fixed on the surface are usually filled with external molecular agents. Two of such possible configurations of the corresponding gadolinium complex on the DND surface, consisting of three carboxy groups, were calculated by the density functional theory method and are shown in Figure 5 for top and side views. Both of these structures correspond to the location of the Gd- ion in the center of isosceles triangles of COO^(−)^ groups on the hydrogenated (111) surface of diamond. Each COO^(−)^ group forms a single covalent bond with gadolinium in both structures. The calculated magnetic moments at the Gd-center are 6.629 *μ*_B_ for the structure shown in Figure 5a,c and 6.523 *μ*_B_ for the structure shown in Figure 5b,d. The calculated values of the magnetic moment correspond to the spin value S = 7/2.

For the configuration 1 in Figure 5a,c the distances between the gadolinium atom and the nearest three oxygen atoms are 0.2281, 0.2182, and 0.2126 nm, respectively. For the triple of these oxygen atoms, the corresponding angles formed by a pair of O atoms and a Gd atom are 114.13°, 131.27°, and 88.32°. The elevation of the Gd^3+^ ion above the (111) surface of diamond is 0.278 nm. Due to the rotational degrees of freedom of carboxy groups around the axes of C-C bonds, with which they are attached to the crystal lattice, various similar configurations of Gd^3+^ chelate complexes can occur at a fixed position of the axes of C-C groups. For such configurations, the elevation of the Gd^3+^ ion above the diamond surface in most cases is approximately the same and is about 0.28 ± 0.05 nm. Configuration 2 of the Gd^3+^ chelate complex, shown in Figure 5b,d and calculated for a closer arrangement of the triad of COO^(−)^ groups in the crystalline sites of the diamond surface, is also typical. For this configuration, the calculated distances between Gd and the three nearest oxygen atoms are 0.2281, 0.2384, and 0.2515 nm. The distances to the other three more distant oxygen atoms are 0.2553, 0.2665, and 0.3588 nm, respectively. The calculated O–Gd–O angles for covalent bonds formed by the three nearest oxygen atoms in the structure under consideration are 116.62°, 110.30°, and 91.82°. In this case, the Gd^3+^ ion rises above the diamond surface to a slightly greater distance of ~0.301 nm. Thus, despite the visible differences in the atomic structure of both chelate complexes 1 and 2, their local environments (Gd–O distances and O–Gd–O angles) are very similar. The average value (0.29 nm) for gadolinium ion elevation obtained by DFT for the considered configurations of chelate complexes is in good agreement with the estimate (0.31 ± 0.03 nm) made by Panich et al. by measuring the spin-lattice relaxation rates of the nuclear magnetic resonance signal from ^13^C nuclei located inside the diamond lattice [24,25]. Such a good agreement between theory and experiment indicates the correctness of the assumptions made about the configurations of the gadolinium chelate complex on the DND surface. 

The similarity in the local environment and Gd–O distances of both configurations 1 and 2 leads to the similarity in the electronic structures of both systems (see Section S1). Formation of the structures shown in Figure 5 provides the appearance of some electronic states on the top of valence bands and bottom of conductive bands (see Figure S1a) related to the localized states in the electronic structure of Gd-centers (see sharp peaks on Figure S1b). Summing up the results of theoretical simulations, it can be argued that the peculiarities of the local arrangement of COO^(−)^ groups on the diamond surface do not affect either the stability of the Gd^3+^ ion in the complex, or the diamond–Gd^3+^ distance, or the electronic structure of the system and Gd- centers.

In the case of eighteen Gd^3+^ ions on the surface of a 5-nm particle, each chelate complex containing a Gd^3+^ ion accounts for an average of 4.3 nm^2^ of the surface area, and the distance between metal atoms is, on average, about 2 nm. Each gadolinium ion can, in principle, be chelated by a pair of carboxy groups, providing additional coordination with other mobile agents, such as water molecules. We also assume that between the nearest gadolinium atoms in chelate complexes there are no bridging …–Gd–O–Gd–… bonds, as well as molecular chains with fragments of the …−Gd−OCO^(−)^−Gd−… type, where gadolinium ions are linked by carboxylate anions and are additionally coordinated by molecular agents from the external environment (for example, water molecules). Nevertheless, a statistically small number of Gd binuclear complexes with gadolinium atoms bound by carboxylate anions can still be present on the surface of DND particles. However, even in this case, for binuclear complexes with a Gd–Gd distance in the range of 0.399–0.466 nm, the antiferromagnetic interaction between the spins of the *4f* shells of gadolinium atoms is negligible [26,27]. At an average distance between metal atoms of ~2 nm, there is no exchange interaction between high-spin agents, and there are no carriers that could transfer it over long distances in the outer molecular shell. Interestingly, a very similar conclusion was made in Ref. [28], where the authors studied the magnetic characteristics of Gd^3+^ ions grafted onto the P^2+^ phosphate groups of DNA molecules in an amount of up to ~20 Gd atoms per one turn of the DNA helix and came to the conclusion that since the average distance between Gd ions is about 0.7 nm, direct exchange interaction between the Gd^3+^ spins cannot exist, and the weak magnetic interaction between the ions is most likely due to the dipole-dipole interaction. 

### 3.3. Electron Paramagnetic Resonance

**Figure 6:**
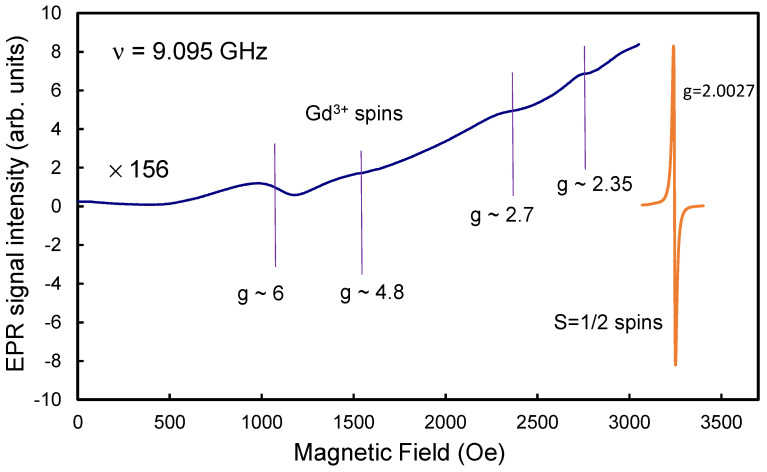
Panoramic EPR spectrum of sample DG4 with the maximum content of gadolinium in the range of 0–3400 Oe. One can see the main EPR signal (g = 2.0027) from diamond nanoparticles and EPR signals from some allowed transitions Δ*m*_s_ = 1 between the levels of the hyperfine structure of the Gd^3+^ ion with magnetic quantum numbers *m*_s_= ±^7^/_2_, ±^5^/_2_, ±^3^/_2_, ±^1^/_2_. Microwave power–1 mW, magnetic field modulation–10 Oe (blue curve) and 0.5 Oe (orange curve), time constant–30 ms.

**Figure 7:**
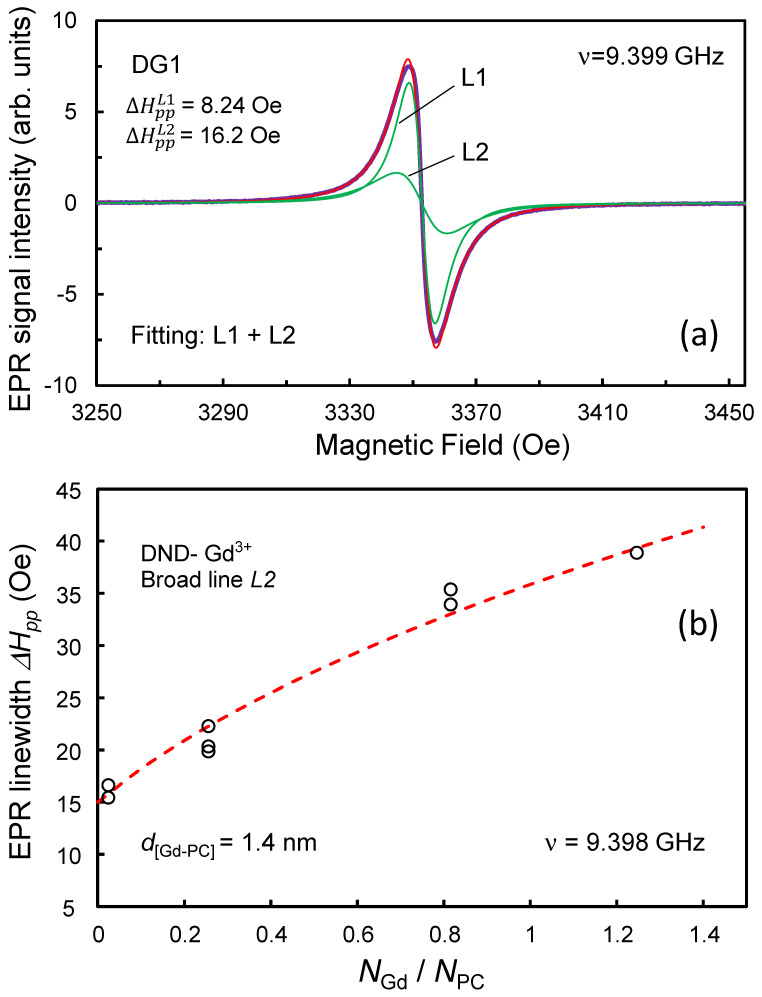
The main EPR signal (*g* = 2.0027) of sample DG1 and its decomposition into two Lorentzian contours *L1, L2* (**a**) together with the dependence of the width of the second component ($∆H_{pp}^{L2}$) on the concentration of gadolinium ions normalized to the concentration of paramagnetic centers (*N*_PC_) of unmodified DND (**b**). The red line in panel (**a**) is the sum of the Lorentzian contours *L1* and *L2* shown in green. The dashed line in panel (**b**) is the fitting curve constructed by Formula (3) with the found optimal parameters. The horizontal axis is *N*_Gd_/*N*_PC_. Microwave frequency–9.399 GHz, microwave power–1 mW, magnetic field modulation–0.5 Oe.

Initially pure DND particles unmodified with *4f*- metals have paramagnetic defects with spin 1/2 in their crystalline matrix, the EPR spectrum of which is characterized by a singlet signal with a *g*-factor of 2.0027 and a width of about Δ*H_pp_* ~ 8.3 Oe. This signal is associated with dangling covalent bonds and other paramagnetic defects, particularly substitutional nitrogen, in the diamond core and was discussed in detail earlier [5,7,14]. A consolidated EPR singlet signal in a DND of such a width is explained by the exchange interaction of the above two groups of spins (by analogy with the conclusions of Ref. [29]), as a result of which each of the spin subsystems loses its individuality. The total concentration of these spins exceeds 1300 ppm. The closeness of the *g*-factor of the DND EPR signal to the same value for a free electron was discussed in one of the first works devoted to the nature of the EPR signal in DND [30] and still continues to be debated. Such a signal consists of two Lorentzian lines-narrow and broad, associated with paramagnetic centers of deep and shallow occurrence, respectively [6]. In this case, the *g*- factors of both lines are approximately the same with an accuracy of ±0.00005, indicating the same nature of the centers of shallow and deep occurrence. The EPR spectrum of DND modified with Gd^3+^ ions, in addition to the recorded broad signal from Gd^3+^ ions extending in a wide range of magnetic fields from zero to 6000 Oe, also demonstrates an intense narrow singlet EPR signal with a *g*- factor of 2.0027, but with a slightly larger (than for pure DND) linewidth Δ*H_pp_*, varying from 8.4 Oe to ~ 12 Oe. As an example, Figure 6 shows a survey (only up to 3400 Oe) EPR spectrum of sample DG4, consisting of an intense singlet *g* = 2.0027 (orange contour) and low-field signals from allowed Δ*m*_s_ = 1 transitions between some of the hyperfine structure levels (*m*_s_ = ±7/2, ±5/2, ±3/2, ±1/2) for Gd^3+^ ions in a magnetic field. Note that in the X-band, EPR signals from Gd^3+^ are very often observed in the range *g* > 2. Signals *g* ~ 6, *g* ~ 4.8 and *g* ~ 2.7 are characteristic markers of the presence of Gd^3+^ ions in different local environments [31,32]. The signal *g* ~ 2 is also characteristic. Similar EPR signals are often observed in various Gd- containing glasses, in which Gd^3+^ ions have different types of local environments and are affected by a low-symmetry crystal/molecular field [32]. We will not discuss here the possible types of the local environment in the vicinity of gadolinium ions on the DND surface, but we note that the presence of an ultra-broad line *g* ~ 2 (in Figure 6, it is intentionally not extended in the region *g* < 2) does not exclude the presence of gadolinium clusters in the system. The nature of the EPR singlet signal (*g* = 2.0027) in all samples DG1–DG4 is the same as for the DND sample not modified with gadolinium, and it also consists of two components. As an example, Figure 7a shows the main EPR signal of the DG1 sample with the minimum concentration of Gd^3+^. It is almost the same as for the original precursor DND, but both of its components of the Lorentzian line shape are slightly broadened. The proximity of Gd^3+^ ions with spin 7/2 to the diamond surface causes a broadening of the DND EPR signal (*g* = 2.0027) associated with dangling bonds and other defects in the crystalline matrix of diamond particles.

For each of the samples of the DND-Gd series, the singlet EPR signal was decomposed into two Lorentzian components (*L1* and *L2*), taking into account additional heuristic considerations about the ratio of the number of shallow (*N*_L2_) and deep (*N*_L1_) centers in particles and their dependence on the number of gadolinium ions per particle surface. (Thus, it was found that as *N*_Gd_ increases, the *N*_L1_/*N*_L2_ ratio increases by 50–100% due to a decrease in the number of near-surface shallow centers). The decomposition of the EPR signal into two Lorentzian contours makes it possible to quite conditionally separate out the contributions to the EPR signal from shallow and deep centers located at different distances from the surface of diamond particles, and thereby track the broadening of each of the Lorentzian signal components due to the external Gd^3+^ paramagnetic agent. The same procedure would formally be valid for a system of three or even more Lorentzian contours, with contributions from paramagnetic centers buried at different distances from the surface, however, the introduction of a larger number of Lorentzian contours to describe the EPR signal in the samples under study is not necessary. Note that the approach to separating paramagnetic centers in DND particles into relatively shallow and deep ones works well, taking into account the relatively wide (not narrow) particle size distribution in the powder (in the range from 3.5 to 7–8 nm), and the different number of paramagnetic centers in individual particles, varying according to their statistical spread. 

The experimentally observed broadening of the DND EPR signal can be well described by Formula (3), both for the narrow and for the broad Lorentzian components:

(3) $${\delta H}_{pp}=\sqrt{{\left(∆H_{pp}^{0}\right)}^{2}+{cn}_{Gd}g^{2}\mu_{B}^{2}S\left(S+1\right)\bar{d_{Gd}^{-6}}}-∆H_{pp}^{0}\approx const\times n_{Gd}\bar{d_{Gd}^{-6}}$$

Here $∆H_{pp}^{0}$ is the width of the EPR line for DND with zero gadolinium content, *n*_Gd_ is the ratio of the number of gadolinium spins (*N*_Gd_) to the number of intrinsic internal spins (*N*_PC_) in DND particles, $d_{Gd}$ is the mean distance between the shallow paramagnetic spin in DND particle and interior Gd^3+^ *4f*- shell spin, and $\bar{d_{Gd}^{-6}}$ is the spatial average for the diamond lattice spin-gadolinium spin distance in the rate −6 for all possible pairs of such spins belonging to one particle; *g* = 1.992 ≈ *2*–*g*-factor for Gd^3+^ EPR signal [33], *μ*_B_ = 9.274 × 10^−21^ erg Oe^−1^ *, S* = ^7^/_2_, *c* = 4/15 × 2.354^2^ ≈ 1.478. Note that the formula is written in the CGS system, where the quantities ${\delta H}_{pp}$ and $∆H_{pp}^{0}$ are given in Oe units, $d_{Gd}$ is given in cm units, and magnetic constant equals $\mu_{0}=1$, thus resulting in the same numerical values for the magnetic field and magnetic flux density (𝐵 = 𝐻) in vacuum. Note: Formula (3) was first obtained in Ref. [34] for the system of spins of triplet oxygen (O_2_) adsorbed on nanographite particles with edge π-electron spins, and then adapted in Ref. [8] for the spins of transition metal ions (copper) on the surface of DND particles with internal paramagnetic defects.

Following this simple formula under the condition ${\delta H}_{pp}$ < (0.4–0.5) $∆H_{pp}^{0}$, the broadening of the Lorentzian components of the EPR signal *g* = 2.0027 (=${∆H}_{pp}^{DND-Gd}$ − ${∆H}_{pp}^{0}$) turns out to be proportional to the Gd^3+^ concentration and the average distance between the Gd^3+^ ion and the paramagnetic center in the DND core to the power of minus 6. In the case of broadenings exceeding the width of the EPR line of a metal-free DND (for each of the EPR signal components-narrow and broad), the ${\delta H}_{pp}$ (*n*_Gd_) dependence smoothly enters a trend with saturation.

With an increase in the concentration of Gd^3+^ ions on the surface of DND particles, the widths of both lines (${\Delta H}_{pp}^{L1}$ and ${\Delta H}_{pp}^{L2}$) from intrinsic DND defects of different localizations increase almost quasi-linearly according to the dipole–dipole interaction mechanism. The narrow line *L1* broadens from 8.2 to 12.8 Oe, while the wider line *L2* broadens from 16 to 39 Oe. However, at large broadenings of the EPR signal, the dependences of ${\Delta H}_{pp}^{L1}$ and ${\Delta H}_{pp}^{L2}$ on *n*_Gd_ are not linear. This is clearly seen for the broad component of the DND EPR signal. This is because at ${\delta H}_{pp}$ > 0.6 $∆H_{pp}^{0}$, the broadening $\delta H_{pp}$ as a function of *n*_Gd_ has a sublinear trend to the extent of the significance of the second term ${cn}_{Gd}g^{2}\mu_{B}^{2}S\left(S+1\right)\bar{d_{Gd}^{-6}}$ under the square root sign in Formula (3). Such a trend can be found for the broad *L*2 line. Figure 7b shows the dependence of the width of the broad component *L*2 of the DND EPR signal associated with shallow centers on the concentration of gadolinium ions *N*_Gd_ normalized to the concentration of intrinsic spin radicals of the unmodified DND sample. The experimental points are well interpolated by the theoretical curve constructed according to Formula (3) using the parameters $∆H_{pp}^{0}$= 15 Oe, *S* = 7/2, $d_{Gd}=d$_[Gd-PC]_ = 1.4 nm. The obtained distance is quite reasonable in magnitude, since the average value of the magnetic field arising at such a distance from the arbitrarily oriented magnetic moment of the gadolinium ion is about $1.5\times 7\mu_{B}/{\left(d_{Gd}\right)}^{3}$ ≈ 35 Oe [35]. This estimate approximately coincides with the EPR linewidth ${\Delta H}_{pp}^{L2}$ (for *n*_Gd_ ≈ 1). Taking into account the fact that, according to the generalized DFT structural model, the Gd ion is located at a distance of ~0.29 nm from the (111) diamond surface, it can be concluded that conventionally shallow paramagnetic centers are located at a distance of 1.1 nm, or at a distance of three lattice constants from the surface. Here we mean the distance corresponding to the centroid of the distribution of centers in depth. At the same time, EPR experiments previously carried out using Cu^2+^ copper ions showed a smaller depth of shallow paramagnetic centers, equal to ~0.76 nm [6]. The reason for this discrepancy can be the more distant (by ~0.39 nm) fixation of Gd^3+^ ions on the diamond surface than for ionic copper, as well as the escape of some electrons (or holes) from very shallow unpaired orbitals to surface sites associated with gadolinium complexes, in as a result of which the former can become non-paramagnetic. Such a mechanism for the disappearance of subsurface paramagnetic centers as a result of electron escape or bond rehybridization in the vicinity of a paramagnetic defect is quite reasonable and was recently described in Ref. [36] to explain the non-paramagnetic status of nitrogen impurities near the diamond crystallite boundary due to surface defects.

Interestingly, in this work, by using intrinsic defects of DND particles as paramagnetic probes, it is possible to establish quantitatively the location of Gd^3+^ ions relative to the former in the range up to ~1.4 nm. This is very similar to the results of a number of modern EPR studies on tracking distances from paramagnetic probes to gadolinium ions in the range of up to ~3.5 nm by recording dipolar-broadened EPR lines [33]. 

The presence of almost perfect paramagnetism in DND–Gd hybrid nanoparticles with a large number (about a couple of tens) of Gd^3+^ spin-7/2 species per particle and Weiss temperature modulo below 0.7 K creates a prerequisite for their consideration as a promising contrast agent for use in MRI instead of the traditionally used Gd^3+^ chelate complexes based on complex organic molecules. At the same time, we note that the physicochemical aspects of the behavior of such particles in solutions and their tendency to aggregation were not the subject of consideration in this article.

## 4. Conclusions

Using SQUID magnetometry, it has been shown that an ensemble of gadolinium ions grafted onto the surface of DND particles through carboxyl groups in an amount of up to 18 per 5-nm particle has remarkable paramagnetic properties, while the antiferromagnetic interaction between spins *S* = 7/2 of Gd^3+^ ions is negligible (the corresponding Weiss temperature is about −0.15…−0.4 K). The average distance between gadolinium ions and shallow DND paramagnetic spin radicals is about 1.4 nm, and the depth of the latter, responsible for the broad Lorentzian component of the DND EPR signal, is about three lattice constants. Magnetic studies indicate the isolated nature of gadolinium chelate complexes on the surface of DND particles and their almost perfect paramagnetism with high concentration (up to 7.9 × 10^19^ g^−1^) of spin-7/2 species.

## Figures and Tables

**Table 1:** Parameters of ensembles of Gd^3+^ ions on the DND surface found from magnetic studies.

Sample Name	$N_{Gd}^{M-H},\;g^{-1}$	$N_{Gd}^{M-H}$, g^−1^ (Corrected)	Δ*T*, K (from *M-H* Curves)	Curie Constant, emu g^−1^ K	$N_{Gd}^{T},g^{-1}$	Θ^Gd^, K (from *χ-T* Curves)	*χ*_o_ ^DND-Gd^, emu g^−1^
DG4	7.85 × 10^19^	7.97 × 10^19^	0.17	1.0146 × 10^−3^	7.76 × 10^19^	−0.33	−0.36 × 10^−6^
DG3	5.14 × 10^19^	5.25 × 10^19^	0.19	6.6024 × 10^−4^	5.05 × 10^19^	−0.08	−0.36 × 10^−6^
DG2	1.61 × 10^19^	1.63 × 10^19^	0.10	2.1626 × 10^−4^	1.65 × 10^19^	−0.69	−0.36 × 10^−6^
DG1	1.53 × 10^18^	1.57 × 10^18^	0.16	2.0380 × 10^−5^	1.56 × 10^18^	−0.49	−0.32 × 10^−6^

## Data Availability

Data are available on request due to restrictions e.g., privacy or ethics.

## Supplementary Materials

The following are available online at https://www.mdpi.com/article/10.3390/nano13131995/s1, Section S1. Calculations of spin-polarized total density of states; Figure S1: Calculated spin-polarized total densities of states (a) and densities of states of Gd centers (b) for two configurations of the gadolinium chelate complex shown on Figure 5 in the main text. Panel (b) also shows the density of states of the Gd center in the hypothetical Gd(OH)_3_ molecule.
