# Supplementary material for: Isolated Spin-7/2 Species of Gadolinium (III) Chelate Complexes on the Surface of 5-nm Diamond Particles
Source: Nanomaterials (Basel). 2023 Jul 1;13(13):1995. doi: 10.3390/nano13131995 (PMC10343492; doi:10.3390/nano13131995)
Supplement: Supplementary file 1 [file nanomaterials-13-01995-s001.zip › nanomaterials-2445483-supplementary.pdf]

Isolated spin-7/2 Species of Gadolinium (III) Chelate Complexes on the Surface of 5-nm Diamond Particles

Vladimir Yu. Osipov [1,*], Danil W. Boukhvalov [2,3] and Kazuyuki Takai [4]

1 Ioffe Institute, Polytechnicheskaya 26, St.-Petersburg 194021, Russia
2 Institute of Materials Physics and Chemistry, College of Science, Nanjing Forestry University, Nanjing 210037, China; danil@njfu.edu.cn
3 Institute of Physics and Technology, Ural Federal University, Mira 19 Str., Yekaterinburg 620002, Russia
4 Department of Chemical Science and Technology, Hosei University, Koganei, Tokyo 184-8584, Japan; takai@hosei.ac.jp
* Correspondence: osipov@mail.ioffe.ru

Content

S1. Calculations of spin-polarized total density of states

The similarity in the local environment and Gd–O distances leads to the similarity in the electronic structures of both systems. Formation of the structures shown in Figure 5 provides the appearance of some states on the top of valence bands and bottom of conductive bands (see Figure S1a) related to the localized states in the electronic structure of Gd-centers (see sharp peaks on Figure S1b). To check the effect of the substrate we performed simulation of hypothetical system $Gd(OH)_3$ where Gd^{3+} is also surrounded by three oxygen atoms. The calculated electronic structure of Gd-center in $Gd(OH)_3$ in the vicinity of Fermi energy is somewhat dissimilar to the electronic structure of the chelate complexes 1 and 2 shown in Figure 5. Based on these results, we can conclude that the presence of the substrate significantly contributes to the electronic structure of Gd-centers. The relative binding energy for the chelate structure 1 shown in Figure 5a,c is only 20 meV lower than for the chelate structure 2 shown in Figure 5b,d.

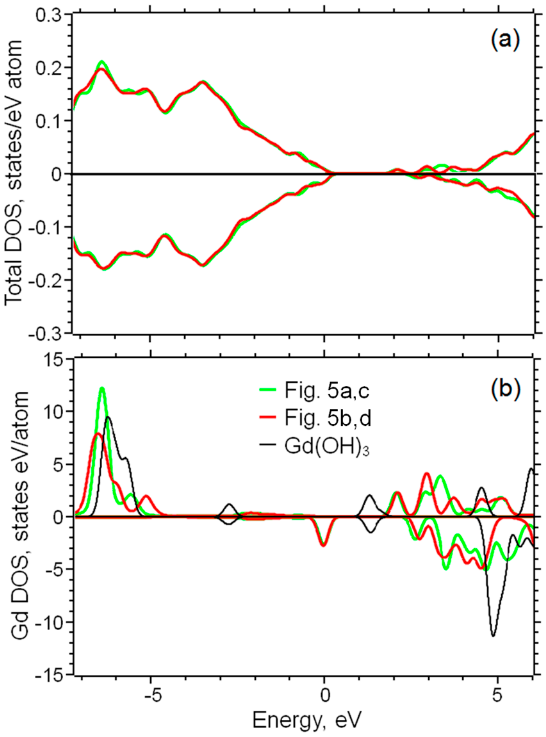

Hence both these chelate structures are equally probable. Summing up the results of theoretical simulations, it can be argued that the peculiarities of the local arrangement of $COO^{(-)}$ groups on the diamond surface do not affect either the stability of the Gd^{3+} ion in the chelate complex, or the diamond-Gd^{3+} distance, or the electronic structure of the system and Gd-centers.

Figure S1. Calculated spin-polarized total densities of states (a) and densities of states of Gd centers (b) for two configurations of the gadolinium chelate complex shown on Figure 5 in the main text. Panel (b) also shows the density of states of the Gd center in the hypothetical $Gd(OH)_3$ molecule.
